# 2171. Minocycline Susceptibility in Carbapenem-Resistant *Acinetobacter baumannii* Blood Isolates in South Korea: Role of *tetB* Gene in Resistance

**DOI:** 10.1093/ofid/ofad500.1793

**Published:** 2023-11-27

**Authors:** Taeeun Kim, Eun Hee Jun, Yoon-Kyoung Hong, Jiwon Jung, Min Jae Kim, Heungsup Sung, Mi-Na Kim, Sung-Han Kim, Sang-Ho Choi, Sang-Oh Lee, Yang Soo Kim, Yong Pil Chong

**Affiliations:** Nowon Eulji University Hospital, Seoul, Seoul-t'ukpyolsi, Republic of Korea; Asan Medical Center, Seoul, Seoul-t'ukpyolsi, Republic of Korea; Asan Medical Center, Seoul, Seoul-t'ukpyolsi, Republic of Korea; Asan Medical Center, Seoul, Seoul-t'ukpyolsi, Republic of Korea; Asan Medical Center, Seoul, Seoul-t'ukpyolsi, Republic of Korea; Asan Medical Center, Seoul, Seoul-t'ukpyolsi, Republic of Korea; Asan Medical Center, Seoul, Seoul-t'ukpyolsi, Republic of Korea; Asan medical center, Seoul, Seoul-t'ukpyolsi, Republic of Korea; Asan Medical Center, Seoul, Seoul-t'ukpyolsi, Republic of Korea; Asan Medical Center, Seoul, Seoul-t'ukpyolsi, Republic of Korea; Asan Medical Center, Seoul, Seoul-t'ukpyolsi, Republic of Korea; Asan Medical Center, Seoul, Seoul-t'ukpyolsi, Republic of Korea

## Abstract

**Background:**

Carbapenem-resistant *Acinetobacter baumannii* (CRAB) represents a growing global threat with minimal therapeutic options. Minocycline (MIN), a semisynthetic tetracycline derivative, has been suggested as an attractive therapeutic option for CRAB infection. However, the optimal dosing and breakpoint for MIN in treating *CRAB* infection remain unclear. Furthermore, nonsusceptibility to MIN may occur through the efflux pump, in particular, TetB. As the prevalence of *tetB* in *A*. *baumannii* has increased, so have the MIN minimum inhibitory concentrations (MICs) for *A. baumannii* strains carrying *tetB*. Here, we evaluated the MIN susceptibility rate in CRAB clinical strains and the association between *tetB* carriage and MIN susceptibility.

**Methods:**

Representative CRAB blood isolates were collected from a tertiary care center in South Korea. The Clinical and Laboratory Standards Institute (CLSI) MIN susceptibility breakpoint for *A. baumannii* was defined as MIC ≤4 mg/L, whereas a new MIN breakpoint of 1 mg/L was suggested based on pharmacokinetic (PK)/pharmacodynamic (PD) studies. For comparison of the impact of the *tetB* resistance, tigecycline was used. Tigecycline susceptibility breakpoint for *Enterobacterales* defined by EUCAST was applied (≤0.5 mg/L). *TetB* carriage was detected by polymerase chain reaction.

**Results:**

Of 160 CRAB isolates, 134 (84%) were susceptible to MIN according to the CLSI criteria, among which 40% (53/134) were PK-PD non-susceptible. The MIN MIC _50/90_ was 1/8 mg/L. Seventy-nine (49%) isolates carried *tetB*. One-third (26/79) of *tetB*-positive and none of the *tetB*-negative isolates were CLSI non-susceptible, while 66% of *tetB*-positive and 33% of *tetB*-negative isolates were PK-PD non-susceptible. *TetB* carriage was correlated with a higher MIN MIC than non-*tetB* carriage (MIC _50/90_ 2/8 mg/L vs. 1/2 mg/L) (Figure 1). However, no clear correlation was observed between *tetB*-positivity and tigecycline MIC (Figure 2). Overall, *tetB* positivity demonstrated 100% sensitivity for MIN CLSI non-susceptibility and 66% sensitivity for PK-PD non-susceptibility.

**Figure:**
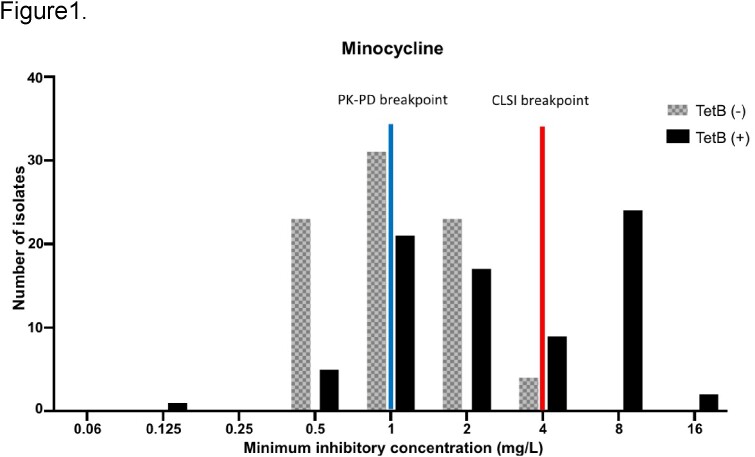

tetB positivity and MICs of minocycline for carbapenem-resistant A. baumannii. The CLSI susceptibility breakpoint for minocycline is ≤4 mg/L, whereas a new MIN PK-PD breakpoint of 1 mg/L was suggested.

Figure2.

**Figure:**
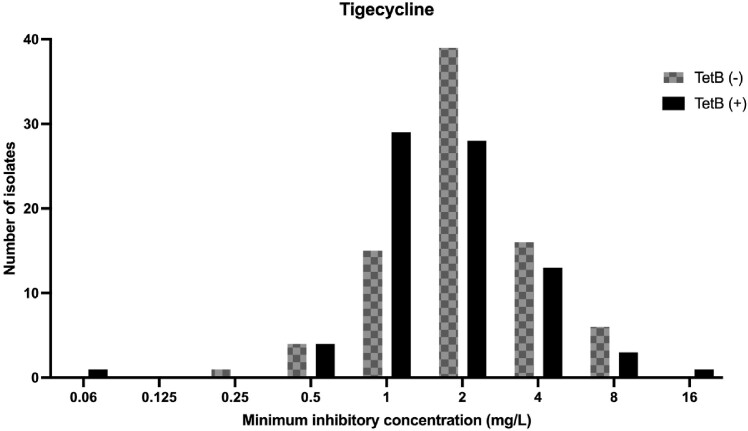

tetB positivity and MICs of tigecycline for carbapenem-resistant A. baumannii. The tigecycline susceptibility breakpoint for Enterobacterales defined by EUCAST was applied (≤0.5 mg/L).

**Conclusion:**

In our study, 49% CRAB isolates carried *tetB* gene. Our findings suggest that the lack of *tetB* is not a reliable marker of MIN PK-PD susceptibility. Therefore, further clinical data on MIN usage is necessary.

**Disclosures:**

**All Authors**: No reported disclosures

